# New insights into the gut-immune-brain axis: immune mechanisms and therapeutic targets for autism spectrum disorder

**DOI:** 10.20517/mrr.2026.48

**Published:** 2026-10-21

**Authors:** John Chulhoon Park, Sin-Hyeog Im

**Affiliations:** ^1^Department of Life Sciences, Division of Integrative Biosciences and Biotechnology, Pohang University of Science and Technology, Pohang 37673, Republic of Korea*.*; ^2^Molecular Neurobiology Laboratory, McLean Hospital and Department of Psychiatry, Harvard Medical School, Belmont, MA 02478, USA.; ^3^Program in Neuroscience, Harvard Medical School, Belmont, MA 02478, USA.; ^4^ImmunoBiome Inc. Bio Open Innovation Center, Pohang 37673, Republic of Korea.

## INTRODUCTION

Autism spectrum disorder (ASD) is a neurodevelopmental disorder that is estimated to affect 1 in 31 children in the United States as of 2025^[1]^. With rising prevalence and lack of effective prevention or treatment strategies, the disorder has captured the attention of the public in recent years. Despite large-scale research, the etiology of ASD remains debated. Genetics is a significant factor in ASD, with up to a thousand different genes involved and a heritability estimate of 80%^[2]^. However, large genetic variability across studies and cohorts suggests that non-genetic factors also contribute to its etiology. Among them, ASD patients are often found to harbor unique gut microbial signatures and immune phenotypes, coalescing to form the theory of a ‘gut-immune-brain axis’ in ASD^[3]^.

### Evidence for a Gut-Microbial Link in ASD

Some of the earliest evidence supporting the gut-brain theory for ASD came from clinical observations of gastrointestinal (GI) disorders, including constipation, diarrhea, and abdominal pain, among patients^[4]^. ASD patients are two- to four-fold more likely to develop GI disorders compared to healthy individuals^[5]^. Notably, the presence of GI disorders is often correlated with higher severity of ASD behavioral symptoms, including repetitive behavior, social withdrawal, irritability, and anxiety^[6,7]^. The gut microbiota is a major regulator of GI disorders, contributing to both the pathogenesis and serving as a potential avenue for treatment^[8]^. It is estimated that 3.8 × 10^13^ bacterial cells from over 3,000 species reside in the human gut^[9]^. These gut microbes closely interact with the host to facilitate digestion, produce essential metabolites, and even regulate the host immune system. Motivated by comorbidity and the recent boom of the ‘microbiome era’, significant efforts have been made to study the ASD gut microbiome. The gut microbiota of ASD patients is often characterized by dysbiosis, with altered diversity and richness compared with neurotypical individuals^[10]^. While specific gut microbial compositions differ from study to study, ASD patients generally have elevated abundances of *Clostridium, Streptococcus, Lactobacillus,* and *Bacteroides* and lower levels of *Bifidobacterium, Akkermansia, Prevotella,* and *Bacteroidetes*^[11-14]^*.* Notably, gut microbial dysbiosis in ASD often shares similarities with signatures observed in inflammatory guts and GI disorders, including elevated *Firmicutes/Bacteroidetes* ratios, lower diversities, and higher abundances of *Lactobacillus, Bacteroides*, and *Escherichia/Shigella*^[15-17]^. However, these phyla and genera comprise vast arrays of bacterial species, and each strain can exert different physiological and immunological effects. Therefore, genera correlations may not accurately reflect the functional properties of each bacterial species, prompting more in-depth studies into the role of distinct gut microbes in ASD.

Microbiome correlations among ASD patients have even spurred causation studies using animal models of ASD. Germ-free (GF) C57BL/6 (B6) mice are characterized by altered behaviors, including stress responses, anxiety, sociability, learning, and motor functions^[18,19]^. In a milestone study, the transfer of fecal microbiota from either neurotypical individuals or ASD patients induced corresponding behavioral phenotypes in GF B6 mice, suggesting that microbiota alone, even in the absence of genetic factors, could result in ASD phenotypes^[20]^. In another study using valproic acid-induced rats, microbiota transplantation regulated short-chain fatty acid (SCFA) and neurotransmitter levels, altering behavior^[21]^.

The gut-brain axis is evident even in genetic models of ASD. For instance, although BTBR mice are genetically predisposed to ASD phenotypes, symptoms are absent in GF-derived BTBR mice^[22]^. Additionally, gut microbiota transplantation from B6 mice confers neurotypical behaviors in BTBR mice, suggesting that the gut microbiome regulates behavior even in the context of ASD genetics^[22]^. In the *Cntnap2* knockout mouse model, social behavior is regulated specifically by the gut microbiota, while host genetics regulates hyperactivity^[23]^. This highlights the unique interplay between ‘nurture’ and ‘nature’ in mouse models of ASD. In both models, microbiome manipulations alter the metabolite pool, suggesting a pathway through which the gut-brain axis may regulate ASD.

Analyses of blood metabolites have revealed elevated levels of lactate, glutamate, histidine, and free fatty acids, among others, in ASD patients^[11,24,25]^. Additionally, a recent longitudinal study of patients found that the majority of the metabolic differences in ASD are accounted for by 14 key biochemical pathways, which are marked by decreases in glutathione, carnosine, 5’-methyltetrahydrofolic acid, and CoQ10, and increases in lactate, glycerol, alanine, threonine, cholesterol, and ceramides^[26]^. Importantly, the gut microbiota is responsible for the production of key neurotransmitters and their precursors, including dopamine, levodopa, tyrosine, serotonin, tryptophan, glutamate, glutamine, and gamma-aminobutyric acid (GABA)^[11]^. Altered levels of some of these neurotransmitters have been observed in ASD patients, including higher levels of glutamate, glutamine, serotonin, and lower levels of GABA^[27-29]^. Although the flow of major gut-derived neurotransmitters is tightly regulated by the blood-brain barrier, their effects can be mediated through both precursors and signaling through the vagus nerve^[30]^. With accumulating metagenomic and correlative data among patients, many studies have hypothesized direct connections between gut microbial metabolites and their functional roles on the brain.

## CRITICISMS OF THE GUT-BRAIN THEORY IN ASD

However, despite growing support for the gut-brain theory, critics have also raised concerns. Concerns have regarded the methodology of human ASD microbiome studies, ranging from sample size, cohort age, ethnicities, and geography, to sample storage and sequencing techniques^[31]^. These differences may often result in inconsistencies in findings across studies^[31]^. For instance, despite large-scale efforts to characterize the ASD microbiome, cross-regional comparisons have often failed to identify significant shared markers. For example, a study comparing ASD cohorts across Shenzhen and Moscow revealed that although 119 to 130 ASD-specific species were identified in each cohort, respectively, only two species, *Eubacterium_sp_CAG_248* and *Prevotella copri,* were found in both^[32]^.

These concerns have prompted debate as to whether the gut microbiota is a consequence, rather than cause, of ASD. For example, dietary intake preferences among ASD patients can, in and of itself, alter these outcomes^[33]^. Compared to neurotypical children, ASD patients were observed to have reduced variety and overall quality of food, with higher intake of simple carbohydrates, lower fruit and vegetable variety, and lower protein intake^[34]^. ASD cohorts have also exhibited greater preference for high-fat/salt and energy-dense foods, with more cheese, processed meats, and oily fish consumption^[35]^. Overall, observable gut microbiome differences in ASD patients can be directly correlated with restricted dietary interests and less-diverse diets^[33]^. However, while these criticisms raise valid concerns about rushing to causal conclusions, they fail to define the directionality in the relationship between the gut microbiota and dietary preferences. More importantly, they also overlook the immunological implications of the gut microbiota and the role of the immune system in ASD.

## THE IMMUNE SYSTEM AS A MEDIATOR OF THE GUT MICROBIOTA AND ASD

The gut microbiota is a fundamental educator of the host immune system. Numerous studies using GF mice have shown major defects in both adaptive and innate immune compartments^[36,37]^. The microbiota can be fine-tuned to poise the immune system towards either a more inflammatory or anti-inflammatory phenotype^[38-40]^. For instance, microbiota-derived SCFAs, including butyrate and propionate, are potent drivers of regulatory T cells (Tregs), while acetate can induce type 1 interferons^[41,42]^. This evidence suggests that changes in the ASD metabolome may elicit functional differences in the host immune system. For example, a longitudinal study of 205 ASD patients identified disruptions in metabolic pathways related to reduced anti-inflammatory and antioxidant defenses^[26]^. Network pharmacology analyses also revealed that, among 51 key genes regulated by microbial metabolites, IL-17, IL-6, and PI3K/Akt pathways were the most affected^[43]^. Notably, the analyses highlight the ability to trace specific metabolites and their producers to distinct physiological pathways, providing greater resolution into understanding how the gut microbiota directly regulates host immune responses during ASD^[43]^.

In line with this background, immune dysfunction itself has been recognized as a major comorbidity of ASD. ASD patients are more likely to develop allergies and autoimmune disorders, such as type 1 diabetes and atopic dermatitis^[44]^. Additionally, plasma cytokine levels of ASD patients are marked by higher levels of proinflammatory interferon (IFN)-γ, interleukin (IL)-1β, IL-6, IL-17, IL-12, and tumor necrosis factor (TNF)-α, while postmortem brain samples have elevated TNF-α, IL-6, IFN-γ, IL-8, and granulocyte-macrophage colony-stimulating factor (GM-CSF) levels^[45-47]^. These observations raise the question of whether inflammation is a causal factor in ASD.

Inflammation during pregnancy is a known risk factor for ASD and is demonstrated by the maternal immune activation (MIA) mouse model. The MIA model utilizes segmented filamentous bacteria (SFB)-mediated IL-17A production to directly elicit ASD-associated behaviors^[48]^. Inflammation-driven ASD is also demonstrated in BTBR mice, which are characterized by heightened peripheral inflammatory markers, as well as greater numbers of inflammatory CD4 and CD8 T cells within the brain^[22,47,49]^. In BTBR mice, suppressing CD4 T cells is sufficient to reduce neuroinflammation and reduce abnormal neuronal activity, resulting in neurotypical behavior^[22]^. Interestingly, these gut-immune dynamics may be partly attributable to links between the intestinal and blood-brain barrier permeability^[50,51]^. For example, gut barrier permeability in BTBR mice directly controls inflammatory cytokine markers within the brain, as well as mouse behavior^[52]^. These emerging studies demonstrate the role of the host immune system in driving ASD in a mouse model. However, further mechanistic studies are required to solidify this gut-immune-brain axis, and translational relevance necessitates analyses of brain-resident T cells from human ASD patient samples to identify their origins and licensing mechanisms.

## NEW PARADIGMS IN NEUROIMMUNE INTERACTIONS

Emerging findings suggest that the effect of inflammatory cytokines on brain function may be more nuanced than previously held^[11]^. For instance, IFN-γ is required for normal social behaviors, and even IL-17A, which causes ASD when elevated prenatally, can improve social behaviors in adult MIA mice^[53,54]^. This dichotomy may be explained by the downstream signals these cytokines elicit. Various neurons express cytokine receptors, including those for TNF-α, IL-1β, IL-6, IL-4, IL-13, and IFN-γ, and each cytokine can prompt distinct neuronal activities^[55]^. For example, basolateral amygdala neurons co-express both IL-17R and IL-10R^[56]^. Administration of IL-17A and IL-17C can induce anxiogenic behaviors by increasing the excitability of these neurons, whereas IL-10 has the opposite effect, demonstrating that specific cytokines can exert distinct outcomes even within the same neurons^[56]^. Cytokine signaling with neurons also affects other ASD-related behaviors, such as social withdrawal. It was recently demonstrated that serotonergic neurons within the dorsal raphe nucleus express IL-1R1^[57]^. IL-1β, including that from the periphery, and lipopolysaccharide induce social withdrawal in mice by acting on these neurons, highlighting another immune axis that regulates social behavior^[57]^.

Furthermore, the recent discovery of brain-resident T cells sheds light on this unique immune-brain link. In recent years, several studies using mouse models have demonstrated causal roles within the gut-immune-brain axis [Figure 1]. In milestone back-to-back publications, brain-resident CD4 T cells were demonstrated to be conduits between the gut and the brain in mice. Large populations of IFN-γ-producing CD4 T cells were identified in the brain parenchyma, localizing within the subfornical organ, brain stem, thalamus, and cerebellum^[58,59]^. Interestingly, these T cells feature nuanced roles that are dependent on the gut microbiota. For example, during homeostasis, IFN-γ-producing T cells are required for normal food-seeking behavior^[58]^. However, IFN-γ and IL-17A can also elicit neuroinflammation and microglial activation when brain-resident T cells are licensed by SFB^[59]^. Taken together, these preclinical studies suggest that brain-resident T cells may be a key to unlocking the gut-immune-brain axis.

**Figure 1:**
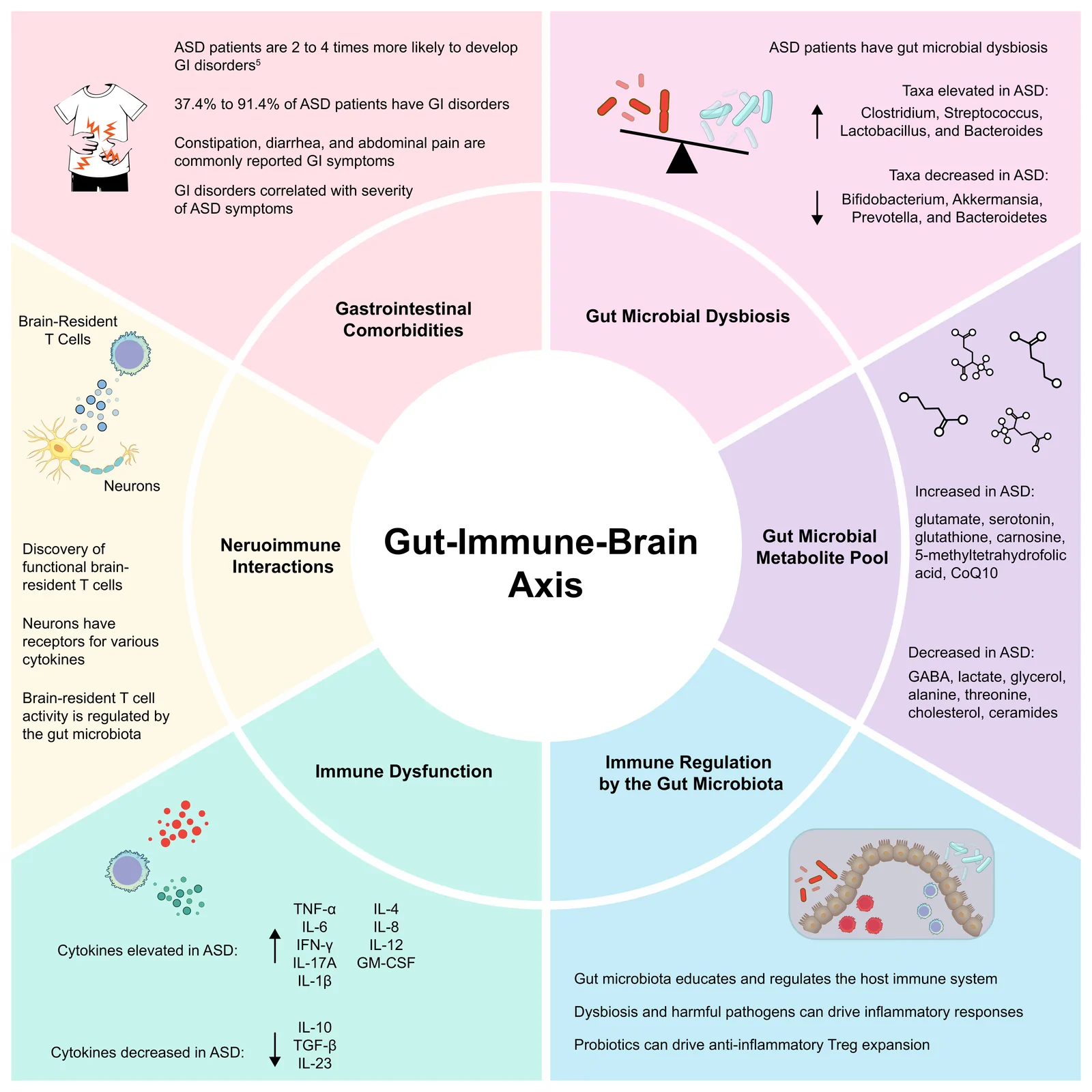
Evidence supporting a gut-immune-brain axis in ASD. ASD patients show comorbidities with gastrointestinal disorders and immune dysregulation, marked by elevated inflammatory cytokines. These clinical observations have prompted extensive investigation into the gut microbial composition in ASD and the associated metabolic signatures. As major regulators of host immunity, the gut microbiota and their metabolites influence peripheral immune responses within the central nervous system, providing a potential mechanistic link between gut dysbiosis and ASD pathophysiology. Created using Adobe Illustrator. ASD: Autism spectrum disorder; TNF-α: tumor necrosis factor-α; IL: interleukin; TGF-β: Transforming growth factor-β; GM-CSF: granulocyte-macrophage colony-stimulating factor; GABA: gamma-aminobutyric acid; IFN-γ: interferon-γ; Treg: regulatory T cell.

## TARGETING THE GUT-IMMUNE-BRAIN AXIS FOR THERAPEUTICS

Theories of a gut-brain etiology in ASD have spurred numerous clinical and preclinical studies that test the efficacy of microbiota-based therapeutics, including probiotics. Across mouse models of ASD, probiotic strains, such as *L. reuteri, Bacteroides fragilis, B. dentium,* and *E. faecalis*, have shown efficacy in improving ASD-related behaviors^[23,60-64]^. However, clinical evidence remains sparse, and certain species used in mouse models, such as *E. faecalis,* may be opportunistic pathogens that require scrutiny when being considered for use in human patients. A few studies have shown mild improvements in ASD patients using consortium probiotics, but these were limited to GI and psychiatric symptoms and partial behavioral phenotypes, and not overall autism severity^[65-67]^.

Major limitations preventing widespread clinical success of probiotics may stem from patient heterogeneity. Diets, immune backgrounds, genetics, and lifestyles influence gut microbial composition and may impact the ability of probiotics to colonize and function within each patient. This suggests that microbiota-based therapeutics require further optimization, including personalized therapies, to improve clinical efficacy. Furthermore, to develop more effective consortium probiotics, the overall metabolic and immunological impact of the consortium should be assessed, as opposed to those of individual species. This will require in-depth analyses of the functional characteristics and interactions among the strains, particularly in the context of each patient’s existing microbiome.

With these considerations, addressing the gut-immune link in ASD may enable more effective therapeutic strategies that target multiple axes simultaneously. For instance, SCFA-producing *Clostridium butyricum* has shown potential to alleviate behavioral abnormalities in BTBR and valproic acid mouse models by enhancing gut barrier function and reducing proinflammatory cytokines^[68]^. Additionally, *L. reuteri*-based probiotic treatment, which can improve the metabolic profile and reduce inflammation, can induce anti-inflammatory Tregs, reduce neuroinflammation, and improve ASD-associated behaviors in BTBR mice^[22]^. In rat models of ASD, administration of *B. longum* and synbiotics, such as the combination of *L. rhamnosus* and luteolin, improves the metabolite pool and lowers proinflammatory cytokine levels within the brain^[69,70]^. These preclinical studies highlight the ability of microbiota-based therapeutics to regulate ASD-associated metabolite pools, reduce inflammation, and thereby ameliorate behavioral outcomes.

## CONCLUSIONS

Uncertainties about the etiology of ASD make it difficult to establish causal directions across pathways, limiting the development of effective therapeutics. However, accumulating data from clinical and animal studies point to the gut microbiota and host immunity as essential components of ASD pathogenesis. Importantly, emerging evidence from animal models indicates that the gut microbiota and its metabolites may act independently of ASD genetics. Immune responses to the gut microbiota and its metabolites may bridge the gut and the brain by regulating neuroinflammation. Future microbiota-based therapeutics should aim to target each component of this axis to achieve greater therapeutic efficacy.

## DECLARATIONS

### Copyright

© The Author(s) 2026.
